# Draft Genome Sequence of a Multidrug-Resistant Clinical Isolate of *Mycobacterium tuberculosis* Belonging to a Novel Spoligotype

**DOI:** 10.1128/genomeA.00965-13

**Published:** 2013-11-21

**Authors:** Shamsudheen Karuthedath Vellarikkal, Ajay Vir Singh, Pravin Kumar Singh, Parul Garg, Viswa Mohan Katoch, Kiran Katoch, Open Source Drug Discovery Consortium, D. S. Chauhan, Sridhar Sivasubbu, Vinod Scaria

**Affiliations:** Genomics and Molecular Medicine, CSIR Institute of Genomics and Integrative Biology, Delhi, Indiaa; GN Ramachandran Knowledge Center for Genome Informatics, CSIR Institute of Genomics and Integrative Biology, Delhi, Indiab; National JALMA Institute of Leprosy and other Mycobacterial Diseases, Tajganj, Agra, Indiac; CSIR Open Source Drug Discovery Unit, Anusandhan Bhavan, New Delhi, Indiad

## Abstract

We describe the genome sequencing and analysis of a multidrug-resistant (MDR) clinical isolate of *Mycobacterium tuberculosis*, strain OSDD105 from India, belonging to a novel spoligotype.

## GENOME ANNOUNCEMENT

Tuberculosis is caused by a closely related group of pathogenic species encompassing the *Mycobacterium tuberculosis* complex. The strains are generally characterized by their distinct spoligotype patterns and grouped into seven major lineages (1). Spoligotype patterns are assayed based on repeat loci in the genome and have been extensively studied in relation to strain lineage, geographical distribution, evolution, virulence, and drug sensitivity. Distinct spoligotypes, including novel spoligotypes, have been previously shown to be associated with specific resistance phenotypes (2–4). Genome sequences of representative members of major genogroups have been reported recently (5–9). The availability of genome sequences of novel spoligotypes would offer a novel opportunity to understand the genome architecture and diversity of these strains and would provide insights into their phenotypic properties. In the present article, we describe the draft genome sequence of a multidrug-resistant (MDR) clinical isolate of *Mycobacterium tuberculosis*, OSDD105, belonging to a novel spoligotype pattern (777737777620000), closely clustering with the Euro-American genogroup.

The clinical isolate OSDD105 was obtained from the strain repository maintained at the National JALMA Institute of Leprosy and other Mycobacterial Diseases, Agra, and was maintained as a part of the Open Source Drug Discovery Open Access Repository. Spoligotyping analysis was performed and drug sensitivity was evaluated per standard protocols (10–12). Analysis revealed that the strain belonged to a novel spoligotype closely clustering to the T2 spoligotype of the Euro-American lineage, with a Spotclust (13) probability of 0.99. Drug sensitivity analysis revealed the isolate to be resistant to streptomycin, rifampin, isoniazid, ethambutol, cycloserine, and pyrazinamide and sensitive to ofloxacin, kanamycin, ethionamide, amikacin, capreomycin, and *para*-aminosalicylate sodium (PAS). DNA was isolated per standard protocols. The raw sequence data were generated after library preparation on Ion Torrent PGM per protocols recommended by manufacturers. Draft genomes were assembled *de novo* using CLC Genomics Workbench 6. The assembly resulted in 181 contigs at *N*_50_ values of 40,206 bp and a total assembly of 4,267,020 bp. Further, automated gene prediction on the draft genomes was performed using the RAST server (14). Analysis revealed 4,760 genes, including 48 RNA genes.

### Nucleotide sequence accession numbers.

This whole-genome shotgun project has been deposited at DDBJ/EMBL/GenBank under the accession AUXD00000000. The version described in this paper is version AUXD02000000.
